# QPSO-Based Adaptive DNA Computing Algorithm

**DOI:** 10.1155/2013/160687

**Published:** 2013-07-15

**Authors:** Mehmet Karakose, Ugur Cigdem

**Affiliations:** ^1^Computer Engineering Department, Firat University, Elazig, Turkey; ^2^Computer Programming Department, Gaziosmanpaşa University, Tokat, Turkey

## Abstract

DNA (deoxyribonucleic acid) computing that is a new computation model based on DNA molecules for information storage has been increasingly used for optimization and data analysis in recent years. However, DNA computing algorithm has some limitations in terms of convergence speed, adaptability, and effectiveness. In this paper, a new approach for improvement of DNA computing is proposed. This new approach aims to perform DNA computing algorithm with adaptive parameters towards the desired goal using quantum-behaved particle swarm optimization (QPSO). Some contributions provided by the proposed QPSO based on adaptive DNA computing algorithm are as follows: (1) parameters of population size, crossover rate, maximum number of operations, enzyme and virus mutation rate, and fitness function of DNA computing algorithm are simultaneously tuned for adaptive process, (2) adaptive algorithm is performed using QPSO algorithm for goal-driven progress, faster operation, and flexibility in data, and (3) numerical realization of DNA computing algorithm with proposed approach is implemented in system identification. Two experiments with different systems were carried out to evaluate the performance of the proposed approach with comparative results. Experimental results obtained with Matlab and FPGA demonstrate ability to provide effective optimization, considerable convergence speed, and high accuracy according to DNA computing algorithm.

## 1. Introduction

DNA computing is a new field of research which performs computing using the biomolecular structure of DNA molecules. The first study performed in the field of DNA computing was the solution of the problem of traveling salesmen composed of 7 cities by Adleman using real DNA molecules [1]. The application was realized by creating solution environment in the biology laboratory and using biochemical reactions. The cities and distances that make up the problem of traveling salesmen were coded using DNA series and all ways that might be solution were created using polymer chain reaction. Upon the success of the study performed a new algorithm for the solution of multivariable problems was acquired. In the continuation of this study Lipton solved satisfiability problem included in NP (nonpolynomial) problem class using DNA computing in a similar way [2]. Lipton demonstrated the application and that DNA computing can be used for the solution of the problems containing logical equations as well. After this study Lin et al. [3] performed the design and optimization of PI (proportional integral) parameters using DNA computing. Ding and Ren used similar DNA computing algorithm with the abovementioned study for setting turbid inspecting parameters [4]. Kim and Lee applied DNA computing algorithm with a different method for setting the PID parameters [5]. In the study performed DNA molecules were used in coding and setting the PI parameters. The results of the application through computer simulation indicated that high success was acquired in this field. Wang et al. compared DNA computers and electronic computers and suggested that DNA computers were more advantageous [6]. As a result of the applications given above, DNA computing was developed rapidly and used in many scientific studies [7–15]. It has been used frequently, particularly in NP problems, coloring problems of graphics in setting the inspecting parameters, arithmetic operations, signal processing problems, and ciphering the data [16–24]. DNA computing algorithm performs computing using the natural characteristics of DNA molecules. Those characteristics include parallel operations, storing high amount of data, providing energy saving, and having significant role in computing. Those characteristics are listed quite effectively in the solution of complex and difficult problems. In order to use the DNA computing more effectively, scientists dealt with the design of total natural DNA computers composed of DNA molecules [25]. 

In Section 2 of this study, DNA computing algorithm is explained in detail. In Section 3 QPSO algorithm is mentioned. In Section 4 DNA computing is made applicable with QPSO algorithm; parameters that increase the effectiveness of DNA computing algorithm are found using QPSO and used for the proposed algorithm. And in the last section the acquired simulation results are given. It is understood from the simulation results that the proposed method produces better values and is more successful.

## 2. DNA Computing Algorithm

**Figure 1:**
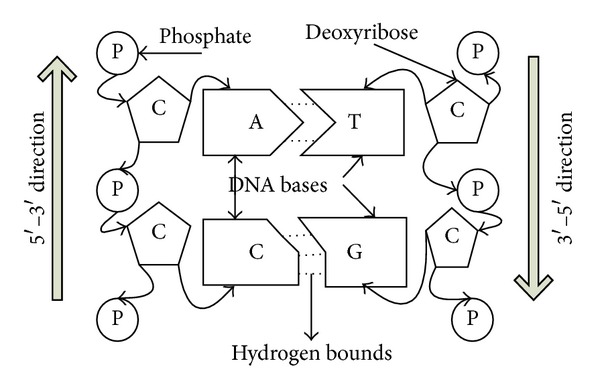
The general structure of the DNA molecule.

DNA computing is a new optimization algorithm performing computing using DNA molecules which store genetic information of the living things. While performing the computing using DNA, DNA bases that make up the ground of the DNA molecules are used. DNA bases are Adenine (A), Guanine (G), Cytosine (C), and Thymine (T) and in this study they have been converted into numerical values including A-0, G-1, C-2, and T-3 [9]. Adenine and Thymine and Guanine and Cytosine complete each other [26–28]. DNA molecules have a structure due to the double and triple hydrogen ties among those bases and in the computing process where number of hydrogen ties is used. In Particular the completion situations of DNA bases and numbers of hydrogen ties are used in the computing performed in solution environments. There are 2 hydrogen ties between Adenine and Thymine and 3 hydrogen ties between Guanine and Cytosine [27]. DNA molecules exist in the form of single serial and double spiral serial. Using the single DNA serials synthesis and reproduction of DNA molecules is realized. Double spiral DNA serials are created according to the Watson-Crick complementation rule. According to this rule Adenine and Thymine and Guanine and Cytosine combine. No emergence is possible. DNA molecules have many advantages including performing parallel operations, storing high amount of data, and using those data for many years without any corruption. These advantages are used sufficiently while performing the computing; the algorithm produces better results. In Figure 1, the general structure of the DNA molecule was shown.

Parallel computing provides a quick conclusion for operations. It is possible to complete the problems that require very big operation volumes and take a long time in the solution with DNA molecules. Some similar problems have many variables and parameters. Coding these variables and parameters and modeling the system generally require data. DNA molecules make great contributions to the solution of these problems with their capability to store data. While DNA computing is applying, the errors are made in the sequence of DNA serials to affect the result negatively. As a result of DNA computing, the values acquired with other optimization algorithm are not deemed to be final results. The studies performed on DNA computing have been implemented in two different manners. The most commonly used method among these is the computing performed in solution environment [26–28]. The cost of performing the computing in solution environment is more because machines are needed to produce DNA serials and DNA synthesis. Furthermore solution should be prepared and solution tubes should be used. Systems named gel electrophoresis are needed for acquiring and analyzing the results [22, 23]. And another algorithm used for DNA computing is the numerical DNA computing algorithm. In this algorithm DNA serials are created in electronic computers and they are converted into numerical values for computing. Although the cost of computing performed electronically is less, the mentioned advantages of DNA molecules cannot be applied on problems completely because when the DNA molecules are used in solution environment, they only can use the abovementioned characteristics.

Numerical DNA computing algorithm is similar to the genetic algorithms; however it is different from genetic algorithms. It uses A, T, G, and C bases rather than dual number of the systems and the solution set of the problems is composed of these bases [29]. Furthermore DNA computing has two new mutation operations. These are enzyme and virus mutations and provide great advantage while computing is performed. Enzyme mutation is the operation of deletion in one or more DNA parts from any DNA serial. And virus mutation is the operation of adding one or more DNA parts to any DNA serial [9, 19, 20]. Enzyme and virus mutations provide continuous renewal of the population and prevent focusing on local optimum points, thus give the algorithm a global search capacity. In Table 1, the conversion of DNA serials into numerical data has been given.

## 3. QPSO Algorithm 

QPSO is an algorithm having the capacity of global searching. This algorithm has been developed inspired by living things that move as masses including insects, bees and were used for the solution of many problems in the literature. In the QPSO algorithm which is a population-based algorithm, individuals act together and compose the masses. While the speed of population is used in PSO algorithm, the next position of the population is determined by taking the average of the best results acquired by the particles that make up the population in QPSO locally. In order to update the speeds and positions of the particles in QPSO algorithm, (1), (2), and (3) are used [30].

While preparing the algorithm the best information acquired by each particle (*p*best) and the best information realized by the mass (*g*best = min (*p*best)) variables are used. Using these two variables the movement point of the mass *P* value is calculated using (1). The *Q*1 and *Q*2 variables in (1) represent random numbers produced in the interval 0-1. In determining the next position of the mass QPSO algorithm uses the variable of *m*best which is the best arithmetic average of mass instead of *g*best. *m*best value is calculated using (2). In (2) *n* represents the population size. After computing the *m*best value for the next step of the population the *x* value is computed by using (3). The *β* variable in (3) is a value to be determined by the applier. And the *u* variable is another one which takes value at the interval of 0-1:

(1) $$\begin{array}{c} p=\frac{\left(Q1*p\text{best}+Q2*g\text{best}\right)}{Q1+Q2}, \end{array}$$

(2) $$\begin{array}{c} m\text{best}=\frac{\left(\sum_{m=1}^{n}p{\text{best}}_{i}\right)}{n}, \end{array}$$

(3) $$\begin{array}{c} x\left(i+1\right)=p\pm \beta \cdot \left|m\text{best}-x\left(i\right)\right|\cdot \ln\left(\frac{1}{u}\right). \end{array}$$

## 4. QPSO-Based Adaptive DNA Computing Algorithm

**Figure 2:**
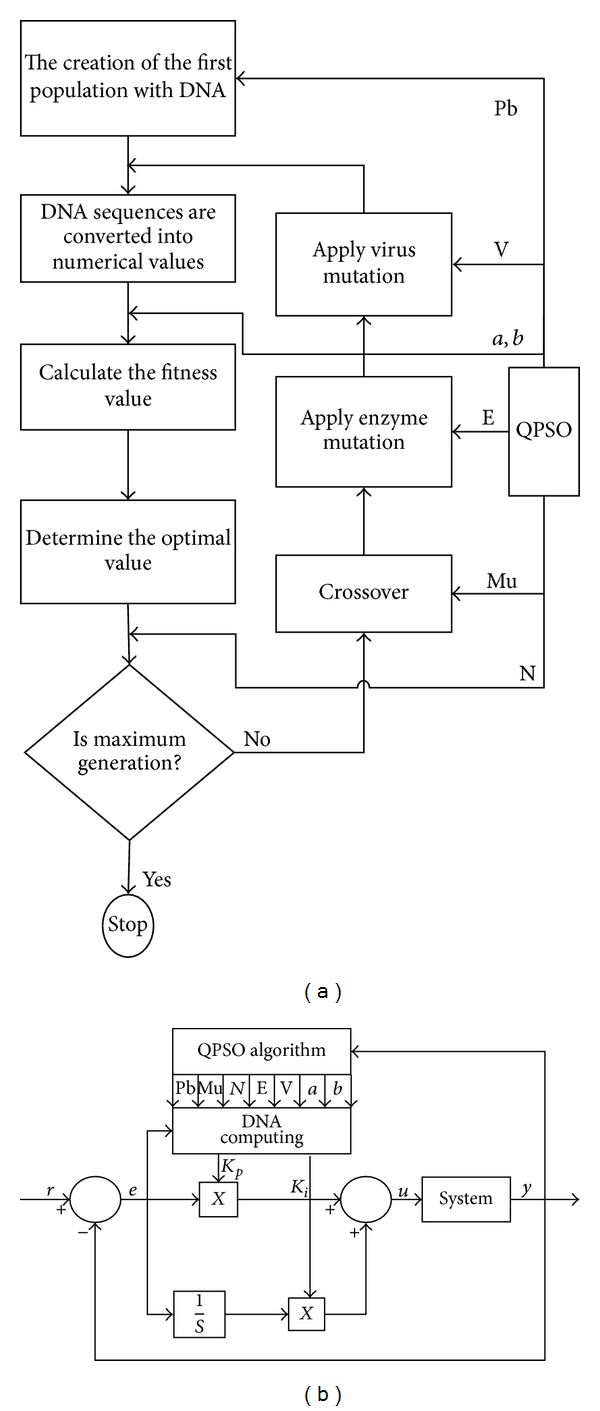
Block diagram of the proposed approach.

In order to increase the global search capacity and effectiveness of DNA computing algorithm in problem solving, parameters should be applicable. The size of the population suitable for this algorithm (Pb), maximum operation number *(N)*, Crossover rate (Mu), enzyme proportion (E), and virus proportion (V) affect the local and global search capacity of the algorithm positively. Making all these parameters adaptable simultaneously can sometimes give negative results. The real target aimed by adapting the parameters is to increase the diversity in the population and prevent the focusing on local optimum points. Furthermore the adaptable algorithms can easily be applied for the solution of many optimization problems. In this study it is aimed to find the optimum values of the parameters considering the conformity values acquired by the population in all iterations of the algorithm. Using the equation depending on conformity function together with the QPSO algorithm, the parameter values are determined. The acquired values are taken as optimized parameter values when optimum solution is achieved. These parameters which are fixed at the beginning are made dynamic and increasing the effectiveness of the algorithm is aimed. In Figure 2, the steps of QPSO based on numerical DNA computing algorithm are shown.

**Figure 3:**
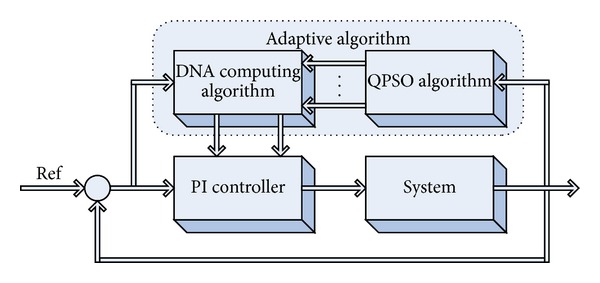
Schematic diagram of the model.

As shown in Figure 3, the proposed approach can be modeled to find the parameters values of DNA computing algorithm using a QPSO algorithm. In the proposed model variables of *K*_*p*_ and *K*_*i*_ are coded with DNA serials at the length of 3 bases. *K*_*p*_ and *K*_*i*_ values are created randomly using A, G, C, and T bases and converted into numerical data using 0, 1, 2, and 3 values, respectively. The serials are converted into first system of 4 then system of 10 and used in numerical environment. The parameter values used for making adaptive DNA computing are explained as follows.

*Population Size (Pb)*. Population size is one of the most significant characteristics that contribute to the solution of the problem. The size of the problem and its complex structure are taken into consideration while determining the value of those parameters. When the population size is increased, a broader solution area is created. The time of solution gets longer. Furthermore unnecessary expansion of the solution area causes big time losses and the solution time for the problem increases rapidly. For this reason optimum value should be selected in the applications. In setting of the population size, (4) is used. The *f* variable given in (4) is the conformity function used for the algorithm. *f*(*n*) gives the conformity value of the *n*th element of the population, and *f*(1) gives the conformity value of the first element of the population. Max(*f*) gives the maximum value of the population and min(*f*) gives the minimum value. These two values provide information about the distribution of the values belonging to population elements. The number 15 given in (4) is the base value and it is used for the purpose of the probability of the size of the population to be zero. It is determined with the method of trial and error:

(4) $$\begin{array}{c} \text{Pop}\_\text{size}=\frac{\left(\max\left(f\right)-\min\left(f\right)\right)}{\left(f\left(n\right)-f\left(1\right)\right)}+15. \end{array}$$

*Maximum Number of Operations (N)*. Maximum number of operations gives the number of processes of the cycle in the DNA computing algorithm. When the maximum number of operations is increased, the completing time of the cycles is extended and the number of the performed operations increases. In Particular the number of elements that composes the population and the number of the variables used in the problem are big; the completion time of the algorithm increases rapidly and this causes loss of time and economic losses. Keeping the maximum number of operations in the lowest level decreases the efficiency of the algorithm. The best values should be determined considering all those situations. In this study, in order to find maximum number of operations, (5) has been used. The number 5 given in (5) is the base value and it is used for the purpose of the probability of the size of the maximum number of operations to be zero and it is determined with the method of trial and error:

(5) $$\begin{array}{c} \text{Maximum}\_\text{generation}\_\text{number}=\left(f\left(n\right)+f\left(1\right)\right)*\text{Pb}+5. \end{array}$$

*Crossover Rate (Mu)*. With Crossover, new elements are created by using the existing population elements. While determining the Crossover rate, the number and selection of the elements to be crossed are taken into account. When the Crossover rate is kept high, the number of elements to be selected increases and the change of the set that shall be created newly is provided. However it is probable that the new created elements may be values close to the parent elements. Furthermore the number of elements to be crossed being high prolongs the duration of completion of the algorithm. In this study (6) has been used for the purpose of finding the Crossover rate. When (6) is used, crossover rate in all iterations is applied at a proportion of 80%:

(6) $$\begin{array}{c} \text{Crossover}\_\text{rate}=\text{Pop}\_\text{size}*0.8. \end{array}$$

*Enzyme (E) and Virus (V) Proportion*. The most significant two parameters used in this study are enzyme and virus mutations. While enzyme mutation provides the deletion of a certain proportion of elements from the population, virus mutation provides the addition of a certain proportion of elements to the population. These two parameters are only used with DNA computing unlike others. The implementing adaptive DNA computing enzyme and virus proportions should be selected well because when the enzyme mutation is not applied in the correct time, it may lead to the loss of elements with good conformity values. One should be careful that the algorithm does not create unnecessary solution area while implementing virus mutation. Equation (7) has been used in the study performed in order to determine enzyme and virus proportions. With the change of the population size, the variation of population at a proportion of 30% is provided in ever iteration. In the selection of the equations found through the trial and error method were taken into consideration and the values acquired by running the system many times were examined in detail and the equations have been given their final forms:

(7) $$\begin{array}{c} \text{e}\_\text{v}=\text{Pop}\_\text{size}*0.3. \end{array}$$

The algorithmic steps of DNA computing algorithm are explained in detail as follows.

**Step 1**

The first population is created with DNA serials randomly.

**Step 2**

The serials created were firstly converted into system of four and then into decimal system as it is explained in Table 3 and converted into numerical data. The operations of coding and converting are given in Table 3 in detail. The population elements converted into numerical data are sent to the simulation environment and the error values created in the system are determined.

**Step 3**

The error values from the simulation are placed in the conformity function and conformity values are obtained. The population elements that minimize sum conformity values are used as the new *K*_*p*_ and *K*_*i*_ values. Equation (8) has been selected for the determination of the conformity value. This conformity function may change in accordance with the preference of the applier as it is explained above.

**Step 4**

The adaptive parameter values to be used for the adaptive DNA computing algorithm and cycle are found using QPSO and sent to the system. Equation (9) is used for the QPSO conformity function. In the selection of the conformity function the results found through the method of trial and error were taken into consideration and the values acquired by running the system many times were examined in detail and the equations have been given their final forms.

**Step 5**

The elements of the population created firstly are subjected to Crossover process and new population is created. Using Steps 2 and 3 new *K*_*p*_ and *K*_*i*_ are determined.

**Step 6**

The population applied enzyme and virus mutation and new population is created. Applying Steps 2, 3, and 4 new *K*_*p*_ and *K*_*i*_ values are determined.

**Step 7**

The best conformity values found in the above steps are compared. *K*_*p*_ and *K*_*i*_ values in the step where the conformity value is minimum are sent to the system.

**Step 8**

Those steps are applied till the maximum number of operations is achieved. At the end of maximum operations the most suitable *K*_*p*_ and *K*_*i*_ values are determined:

(8) $$\begin{array}{c} f\left(K_{p},K_{i}\right)=\text{sum}\left(\text{abs}\left(e\right)\right), \end{array}$$

(9) $$\begin{array}{c} f\_\text{qpso}=a*\text{sum}\left(e^{2}\right)+b*\max\left(\text{yout}\right), \end{array}$$

(10) $$\begin{array}{c} a=\text{average}\left(f\_\text{qpso}\right)*c1, \end{array}$$

(11) $$\begin{array}{c} b=\text{average}\left(f\_\text{qpso}\right)*c2. \end{array}$$

## 5. Experimental Results

A transfer function given with equation (12) that is modeling position control of a DC motor was used for the performance measurement of the proposed method. DNA computing algorithm was applied for setting the PI parameters and Matlab m-file was written. For finding the simulation results, the model given in Figure 3 was created in the Simulink environment and the results were obtained. The parameter values used for DNA computing are given in Table 3:

(12) $$\begin{array}{c} G\left(s\right)=\frac{2.2}{8.96e-6s³+7.27e-3s²+0.945s}. \end{array}$$

**Figure 4:**
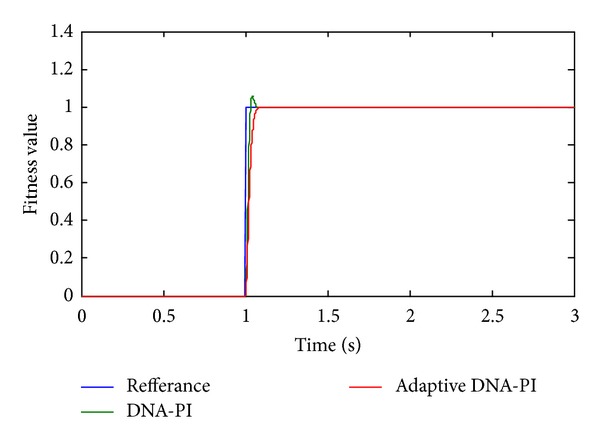
Adaptive algorithm results.

In the application performed, QPSO-based DNA computing algorithm was used for the optimization of the PI parameters. Although various conformity functions were used in the optimization of the PI parameters, in this study the sum of absolute value of error was selected as the conformity function. With the use of this function which is sensitive even to the errors with minimal values it was targeted that upper excess, increase, and setting times would give better results. In the Matlab/Simulink study performed the size of the population used in the application was taken as 80, maximum number of operations as 20, and reference value as 1. For the detection of *K*_*p*_ and *K*_*i*_ values (8) has been used as the conformity function. While performing coding with DNA computing algorithm *K*_*p*_ and *K*_*i*_ values were coded with DNA basis using data of 6 bytes. Firstly 80 individuals in the population were used and each individual was represented with data of 12 bytes. The first 6 bytes of those data of 12 bytes were used for *K*_*p*_ and the other 6 bytes were used for *K*_*i*_. In every iteration enzyme and virus mutation as much as 30% of the population was applied and the change of the individual was provided as much as population size ∗ 0.3 and the population was renewed. In the application performed, the population elements created in the algorithms were sent to the system and determination of the PI elements has been provided. The results produced by the system as a result of running the program many times are given in Table 3 and Figure 4.

As it is given in Table 2, using the adaptive DNA computing algorithm *K*_*p*_ is found to be 17, *K*_*i*_ 0.4375, placement time 0.08 seconds, and maximum excess approximately 0%. In Figure 4, the comparison of the results found with adaptive DNA computing to DNA computing is given. The maximum excess value found with DNA computing algorithm made adaptive is approximately 0% while the maximum excess value found with DNA computing is computed as approximately 14%. Those results indicate that adaptive DNA computing gets better results.

**Figure 5:**
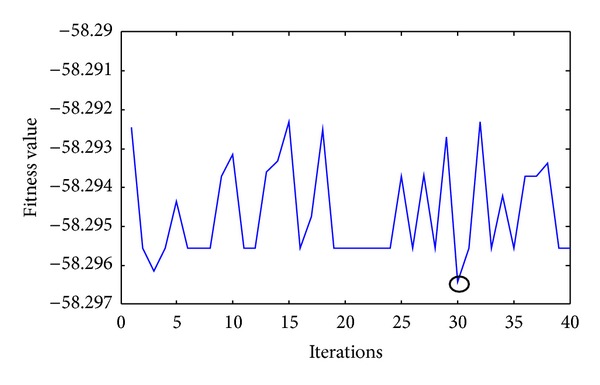
The fitness values of adaptive DNA computing algorithm.

**Figure 6:**
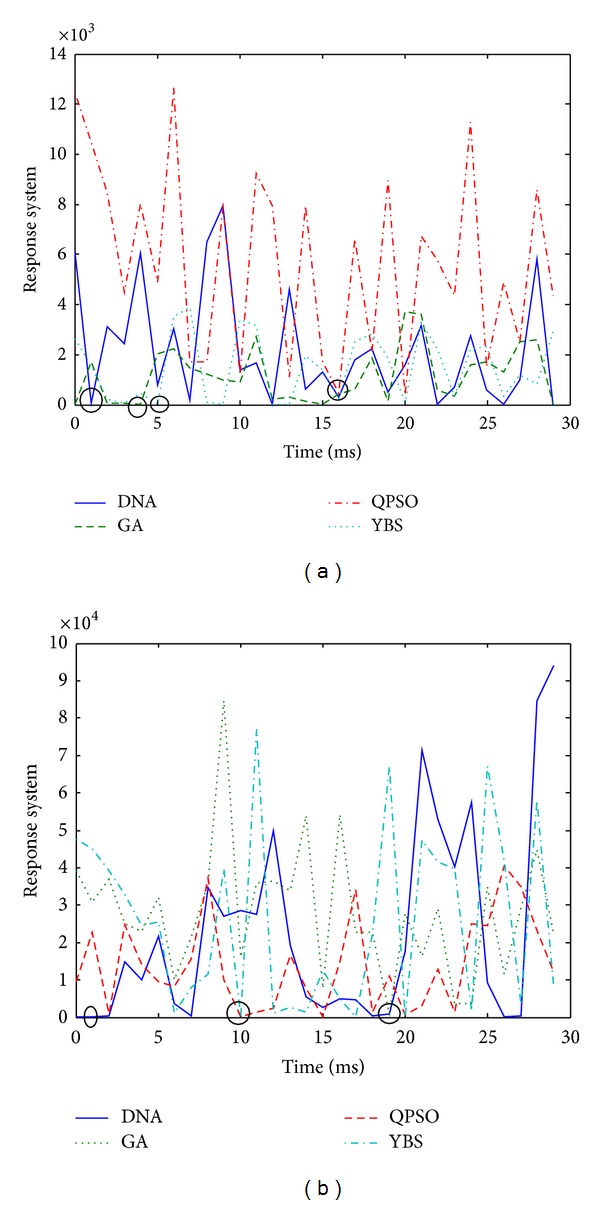
Comparison results.

In Table 3 DNA computing parameters made adaptive with the QPSO algorithm and DNA computing parameters are given. With the activation of the algorithm with QPSO parameter values have changed. With the QPSO algorithm population size was revised as 60, maximum number of operations as 40, Crossover rate as 32%, and enzyme and virus proportion as 12%. New values were applied by being sent to DNA computing algorithm. The values of *a* and *b* used for the conformity function were 15 and 12 when the algorithm started and with the QPSO algorithm they were acquired as 11.8203 and 1.9700 and used for the system. The performance of the proposed approach and comparison results are shown in Figures 5 and 6, respectively. As shown in these figures, the proposed approach finds faster than other algorithms.

## 6. Conclusions

DNA computing that the key advantage is parallelism has the natural capabilities of DNA and large information storage abilities. But this computation model has some problems for numerical implementation in real world applications. In particular, the parameters of the DNA computing algorithm are determined by trial and error in the literature. This determination method is usually very difficult, inefficient, and inflexible. A new QPSO-based adaptive DNA computing algorithm is proposed and implemented for optimization problems in this study. The seven parameters of DNA computing algorithm has been simultaneously and online tuned with QPSO for adaptive operation in the proposed approach. Experimental results obtained with computation of parameters of PI controller for system identification application consistently show that proposed approach has effective optimization, high accuracy and high convergence speed according to traditional DNA computing algorithm. In addition, general search capability of the method is not dependent on local optimum points and applicability of the proposed approach is easier, simpler, and faster than traditional DNA computing algorithm.

## Figures and Tables

**Table 1:** Coding table of DNA computing algorithm.

	DNA coding system	Quartet coding system	Binary coding system	Decimal coding system	Minimum value of *K*_*p*_ and *K*_*i*_	Maximum value of *K*_*p*_ and *K*_*i*_
	A, G, C, T	0, 1, 2, 3	00, 01, 10, 11			
*K*_*p*_	AGT, TAA, TTT	013, 300, 333	000111, 110000, 111111	0 ∗ 16 + 1 ∗ 4 + 3 ∗ 1 = 7 3 ∗ 16 + 0 ∗ 4 + 0 ∗ 1 = 48 3 ∗ 16 + 3 ∗ 4 + 3 ∗ 1 = 63	0	63
*K*_*i*_	GTT, CCA, AGC	133, 220, 012	011111, 101000, 000110	1 ∗ 1 + 3 ∗ 1/4 + 3 ∗ 1/16 = 1.9375 2 ∗ 1 + 2 ∗ 1/4 ∗ 0 ∗ 1/16 = 2.5 0 ∗ 1 + 1 ∗ 1/4 + 2 ∗ 1/16 = 0.375	0	4

**Table 2:** Comparison results of DNA and adaptive DNA computing algorithms.

Algorithm	*K*_*p*_	*K*_*i*_	Settling time	Overshoot %
DNA-PI	30	0.1250	0.08	14
Adaptive DNA-PI	17	0.4375	0.08	~0

**Table 3:** Values of DNA and adaptive DNA computing parameters.

Parameters	DNA computing	Adaptive DNA computing
Population size	80	60
Maximum generations	20	40
Crossover rate	%100	%32
Enzyme and virus rate	%30	%12
*a * and *b *	15, 12	11.8203, 1.9700

## References

[1] Adleman LM (1994). Molecular computation of solutions to combinatorial problems. *Science*.

[2] Lipton RJ (1995). Using DNA to solve NP-complete problems. *Science*.

[3] Lin C-L, Jan H-Y, Hwang T-S Structure variable PID control design based on DNA coding method.

[4] Ding Y, Ren L DNA genetic algorithm for design of the generalized membership-type Takagi-Sugeno fuzzy control system.

[5] Kim JJ, Lee JJ (2008). PID controller design using double helix structured DNA algorithms with a recovery function. *Artificial Life and Robotics*.

[6] Wang Y, Cui G, Wang Z Research on DNA computer with coprocessor organizational model.

[7] Zhang X, Niu Y, Wang Y DNA computing in microreactors: a solution to the minimum vertex cover problem.

[8] Sridhar R, Balasubramaniam S GIS integrated DNA computing for solving travelling salesman problem.

[9] Çiğdem U, Karaköse M Using of DNA computing for tuning of parameters of PI and fuzzy controllers.

[10] Li F, Li Z, Xu J DNA computing model based on photoelectric detection system with magnetic beads.

[11] Huang Y, He L DNA computing research progress and application.

[12] Xu J, Qiang X, Yang Y (2011). An unenumerative DNA computing model for vertex coloring problem. *IEEE Transactions on Nanobioscience*.

[13] Mitra S, Das R, Hayashi Y (2011). Genetic networks and soft computing. *IEEE/ACM Transactions on Computational Biology and Bioinformatics*.

[14] Jiao H, Zhong Y, Zhang L, Li P Unsupervised remote sensing image classification using an artificial DNA computing.

[15] Yin Z, Hua C, Song B Plasmid DNA computing model of 0-1 programming problem.

[16] Xu J, Qiang X, Zhang K A parallel type of DNA computing model for graph vertex coloring problem.

[17] Huang Y, Yin Z, Tian Y Design of PID controller based on DNA computing.

[18] Yin Z, Song B, Zhen C, Hua C Molecular beacon-based DNA computing model for maximum independent set problem.

[19] Lin C-L, Jan H-Y, Hwang T-S Structure variable PID control design based on DNA coding method.

[20] Lin C-L, Jan H-Y, Huang T-H Self-organizing PID control design based on DNA computing method.

[21] Henkel CV (2005). *Experimental DNA computing [Ph.D. thesis]*.

[22] Qiu ZF (2003). *Advance the DNA computing [Ph.D. thesis]*.

[23] Rahman MO, Hussain A, Scavino E, Hannan MA, Basri H Object identification using DNA computing algorithm.

[24] Chaves-González JM, Vega-Rodríguez MA DNA sequence design for reliable DNA computing by using a multiobjective approach.

[25] Zhang H, Liu X A general object oriented description for DNA computing technique.

[26] Jiao H, Zhong Y, Zhang L (2012). Artificial DNA computing-based spectral encoding and matching algorithm for hyperspectral remote sensing data. *IEEE Transactions on Geoscience and Remote Sensing*.

[27] Liu W, Sun S, Guo Y A DNA computing model of perceptron.

[28] Zhang Q, Xue X, Wei X (2012). A novel image encryption algorithm based on DNA subsequence operation. *The Scientific World Journal*.

[29] Xu J, Qiang X, Yang Y (2011). An unenumerative DNA computing model for vertex coloring problem. *IEEE Transactions on Nanobioscience*.

[30] Xi M, Sun J, Xu W (2007). Parameter optimization of PID controller based on Quantum-Behaved Particle Swarm Optimization Algorithm. *Complex Systems and Applications-Modelling*.

